# Pulsatilla Saponins Inhibit Experimental Lung Metastasis of Melanoma via Targeting STAT6-Mediated M2 Macrophages Polarization

**DOI:** 10.3390/molecules28093682

**Published:** 2023-04-24

**Authors:** Xin Yang, Miaolin Wu, Xin Yan, Cheng Zhang, Yingying Luo, Jun Yu

**Affiliations:** 1 Center for Translational Medicine, Jiangxi Key Laboratory of Traditional Chinese Medicine in Prevention and Treatment of Vascular Remodeling Associated Disease, Jiangxi University of Chinese Medicine, Nanchang 330006, China; 2 The Second Affiliated Hospital, Jiangxi University of Chinese Medicine, Nanchang 330006, China; 3 Department of Cardiovascular Sciences and Center for Metabolic Disease Research, Lewis Katz School of Medicine, Temple University, Philadelphia, PA 19140, USA; 4 State Key Laboratory of Innovative Drug and Efficient Energy-Saving Pharmaceutical Equipment, No. 56 Yangming Road, Nanchang 330006, China

**Keywords:** *Pulsatilla saponins*, M2 macrophages, STAT6, tumor metastasis

## Abstract

*Pulsatilla saponins* (PS) extracts from Pulsatilla chinensis (Bge.) Regel, are a commonly used traditional Chinese medicine. In the previous study, we found *Pulsatilla saponins* displayed anti-tumor activity without side effects such as bone marrow suppression. However, the mechanism of the anti-tumor effect was not illustrated well. Since M2-like tumor-associated macrophages (TAMs) that required activation of the signal transducer and activator of transcription 6 (STAT6) for polarization are the important immune cells in the tumor microenvironment and play a key role in tumor progress and metastasis, this study aimed to confirm whether *Pulsatilla saponins* could inhibit the development and metastasis of tumors by inhibiting the polarization of M2 macrophages. We investigated the relevance of M2 macrophage polarization and the anti-tumor effects of *Pulsatilla saponins* in vitro and in vivo. In vitro, *Pulsatilla saponins* could decrease the mRNA level of M2 marker genes Arg1, Fizz1, Ym1, and CD206, and the down-regulation effect of phosphorylated STAT6 induced by IL-4; moreover, the conditioned medium (CM) from bone marrow-derived macrophages (BMDM) treated with *Pulsatilla saponins* could inhibit the proliferation and migration of B16-F0 cells. In vivo, *Pulsatilla saponins* could reduce the number of lung metastasis loci, down-regulate the expression of M2 marker genes, and suppress the expression of phosphorylated STAT6 in tumor tissues. Furthermore, we used AS1517499 (AS), a STAT6 inhibitor, to verify the role of PS on M2 macrophage polarization both in vitro and in vivo. We found that *Pulsatilla saponins* failed to further inhibit STAT6 activation; the mRNA level of Arg1, Fizz1, Ym1, and CD206; and the proliferation and migration of B16-F0 cells after AS1517499 intervention in vitro. Similar results were obtained in vivo. These results illustrated that *Pulsatilla saponins* could effectively suppress tumor progress by inhibiting the polarization of M2 macrophages via the STAT6 signaling pathway; this revealed a novel mechanism for its anti-tumor activity.

## 1. Introduction

Tumor-associated macrophages (TAMs) are one of the main immune cells in the tumor microenvironment and play critical roles in cancer development [1]. Recruited by various cytokines in the tumor microenvironment, peripheral blood mononuclear cells infiltrate into tumors and differentiate into TAMs [2,3]. TAMs can be divided into two major types: classically activated macrophages (M1) and alternatively activated macrophages (M2). M1 macrophages are mainly involved in the Th1-type immune response by killing tumor cells and pathogens, including bacteria and viruses [4,5]. On the other hand, M2 macrophages are primarily involved in angiogenesis, tissue remodeling, wound healing, anti-inflammatory activity, and T cell immune function regulation by secreting various cytokines and chemokines, including IL-10, Arg1, Fizz1, Ym1, CD206, and low levels of IL-12 [6,7]. Most TAMs present the M2 phenotype and promote metastasis, angiogenesis, and immune suppression [8,9,10,11,12]. Thus, the inhibition of M2 macrophage polarization may be a potential target for cancer immunotherapy.

Melanoma is a skin cancer caused by the malignancy of melanocytes [13,14]. The incidence of melanoma is rapidly increasing worldwide, which results in public health problems. The typical therapy for malignant melanoma is surgical excision, immunotherapy such as gene therapy, and chemotherapy [15,16]. Currently, a massive in vivo recruitment of M2-polarized macrophages was observed in human and murine melanoma models injected in mice [17,18]. Thus, M2-polarized macrophages are a practical entry point for the treatment of melanoma.

The signal transducer and activator of transcription 6 (STAT6) is a member of the STAT family, which plays a crucial role in regulating cell differentiation, cytokine production, and promoting macrophage M2 polarization. Interleukin-4 (IL-4) binds to the IL-4 receptor and GP130 and phosphorylates STAT6. Following homodimerization, phosphorylated STAT6 undergoes nuclear translocation, and the transcription activation of genes that initiate M2 macrophage polarization [19]. Previous studies have shown that STAT6 is constitutively activated during cancer development [20]. The inhibition of STAT6 phosphorylation impairs the proangiogenic effects of M2 macrophages [21].

*Pulsatilla saponins* (PS) are the saponin fraction of extracts from *Pulsatilla chinensis* (Bge.) Regel, a traditional Chinese medicine known for its significant anti-inflammatory effects. Studies from our team have shown that PS inhibited tumor growth by promoting tumor cell apoptosis, inhibiting cell proliferation, and disturbing tumor energy metabolism in vivo and in vitro [22,23]. Several effective anti-tumor constituents have been identified from the PS fraction, such as Pulsatilla saponin D (PSD), Raddeanoside R13 (R13), and Pulsatilla saponin A (PSA), etc. Our previous study revealed that a combination of PSD, R13, and PSA had a synergetic effect in inducing tumor cell apoptosis [24]. The same effect has been shown in hepatoma H22-bearing mice. The tumor inhibition rate of Pulsatilla saponins was 71.3%, but the inhibition rates of PSD, R13, and PSA were 57.7%, 62.1%, and 49.2%, respectively. These results suggest that PS is more effective in inhibiting tumors in vivo [25,26], but the mechanism is unclear. The present study demonstrates that PS inhibited B16-F0 melanoma growth and metastasis by attenuating M2 macrophage polarization via the STAT6 signaling pathway.

## 2. Results

### 2.1. PS Inhibits Macrophage M2 Polarization by Inhibiting IL-4/STAT6 Signal Pathway

**Figure 1:**
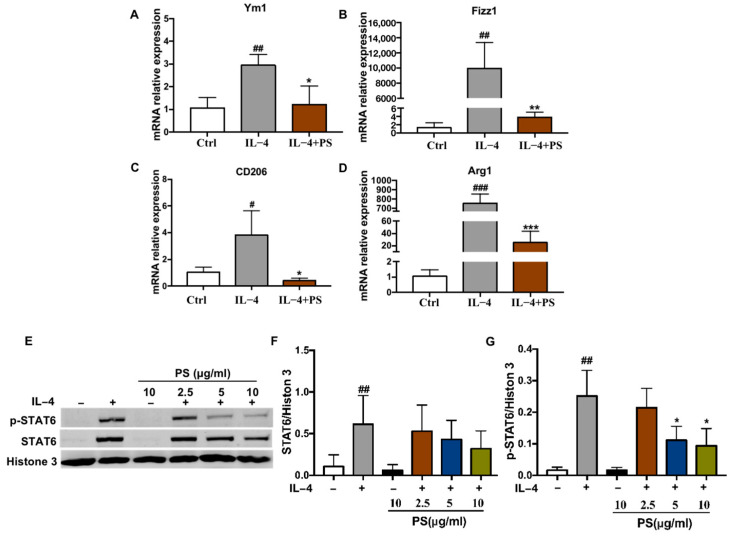
*Pulsatilla saponins* inhibit M2 macrophage polarization. (**A**–**D**) The mRNA levels of Ym1, CD206, Fizz1, and Arg1 were detected by real-time qPCR. (**E**) STAT6 and phosphor-STAT6 protein in nuclear lysates of BMDMs detected by Western blot analysis. Histone 3 as a loading control. (**F**) STAT6 protein quantified with Histone 3 normalization. (**G**) The phosphor-STAT6 protein quantified with Histone 3 normalization. Data were presented as mean ±  SD. *# p  < * 0.05, *## p  < * 0.01, *### p  < * 0.001 compared with control group; * p < 0.05, ** p < 0.01, *** p < 0.001 compared with IL-4 group. All experiments were repeated 3 times.

To investigate whether PS may alter macrophage polarization, we utilized an M2 macrophage activation experiment by IL-4 stimulation in vitro. Bone marrow-derived macrophages (BMDMs) from C57Bl/6 mice were pre-treated with PS (2.5, 5, 10 μg/mL) or a vehicle for 12 h, and then stimulated with IL-4 (25 ng/mL) for 6 h, and all cells were collected after treatment for qPCR. As shown in Figure 1A–D, the levels of M2 macrophage marker genes Arg1, Fizz1, Ym1, and CD206 mRNA were significantly increased after IL-4 stimulation in BMDM, suggesting Il-4 could induce macrophage M2 polarization. PS substantially down-regulated Arg1, Fizz1, Ym1, and CD206 mRNA levels, compared to IL-4 stimulation alone, indicating that PS can inhibit M2 macrophage polarization. Published data have shown that cytokines such as IL-4 activate STAT6, and phosphorylated STAT6 translocates to the nucleus, and promotes transcription of M2 macrophage marker genes. Therefore, we further investigated the protein level of phosphor-STAT6 in nuclei. Except for the Ctrl group, BMDMs were pre-treated with PS (2.5, 5, 10 μg/mL) or the vehicle for 6 h, then stimulated with 25 ng/mL IL-4 for 15 min; all cells were collected after treatment for a Western blot analysis. As shown in Figure 1E–G, the level of STAT6 and phosphor-STAT6 was significantly increased in nuclei after IL-4 stimulation, and PS substantially decreased the phosphor-STAT6 level, not the STAT6 level. PS did not affect BMDMs without IL-4 stimulation (Figure 1E, lane 3). Taken together, these results suggest PS can inhibit M2 polarization, and attenuate STAT6 phosphorylation and translocation in BMDMs.

### 2.2. Conditioned Medium (CM) from M2 Macrophage Treated with PS Inhibits B16-F0 Cell Proliferation and Migration In Vitro

**Figure 2:**
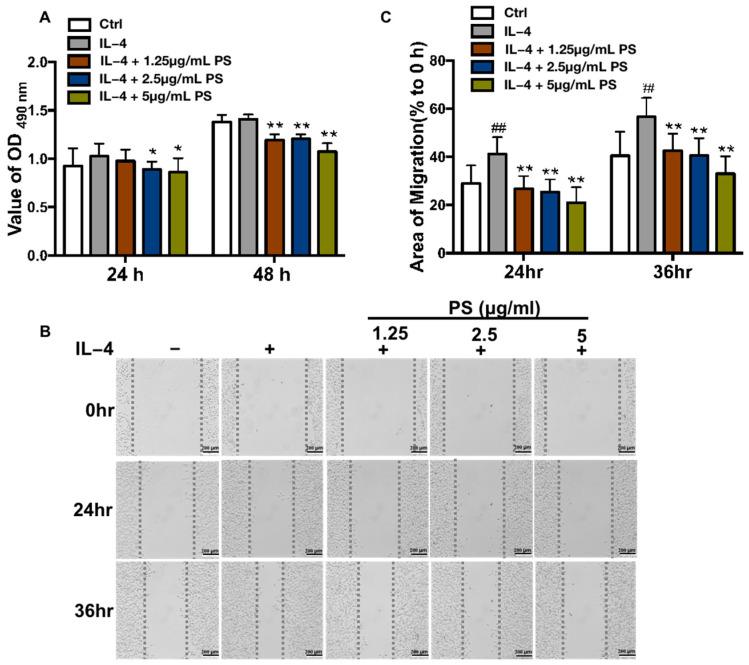
Pulsatilla saponins inhibit melanoma cell migration by suppressing M2 macrophage polarization. (**A**) Effects of BMDM-conditioned medium on the cell proliferation of B16-F0 cells by MTT assay; (**B**) representative images for wound-healing assays. Images were captured at 0, 24, and 36 h. (**C**) Quantification for wound-healing assay. The percentage of the area covered by the migrating cells was measured by using Image J. Data were presented as mean ± SD. ## *p*  <  0.01, compared with control group; * *p* < 0.05, ** *p* < 0.01, compared with IL-4 group. All experiments were repeated 3 times.

As we all know, M2-polarized macrophages can promote tumor cell proliferation and migration through paracrine effects [27]. To examine whether PS affects the proliferation and migration of tumor cells by inhibiting macrophage polarization, a conditioned medium (CM) was collected from IL-4-stimulated BMDMs pretreated with the vehicle (IL-4-CM) or PS (IL-4+PS-CM), and CM from common culture BMDM_S_ without IL-4 or PS treatment acted as the control (Ctrl-CM). Cell proliferation of B16-F0 was tested by MTT, after being treated with different CM for 24 and 48 h. As a result, as is shown in Figure 2A, compared with Ctrl-CM, IL-4-CM had no effect on B16-F0 proliferation, but IL-4+PS-CM significantly inhibited B16-F0 proliferation compared with IL-4-CM (Figure 2A). Cell migration is a crucial event in many biological processes, including cancer progression, tissue formation, and immune defense [28]. The wound-healing assay is a common method to observe the migration ability of cancer cells [29]. The results of the wound healing experiment showed that, compared to Ctrl-CM, IL-4-CM significantly promoted the migration of B16-F0 cells. Furthermore, IL-4+PS-CM notably inhibited B16-F0 migration compared with IL-4-CM from IL-4 (Figure 2B,C). These results indicated that PS inhibited B16-F0 cell proliferation and migration, which was related to its effect on inhibiting M2 polarization.

### 2.3. PS Inhibits Macrophage M2 Polarization on Tumor Cells in a STAT6-Dependent Manner

**Figure 3:**
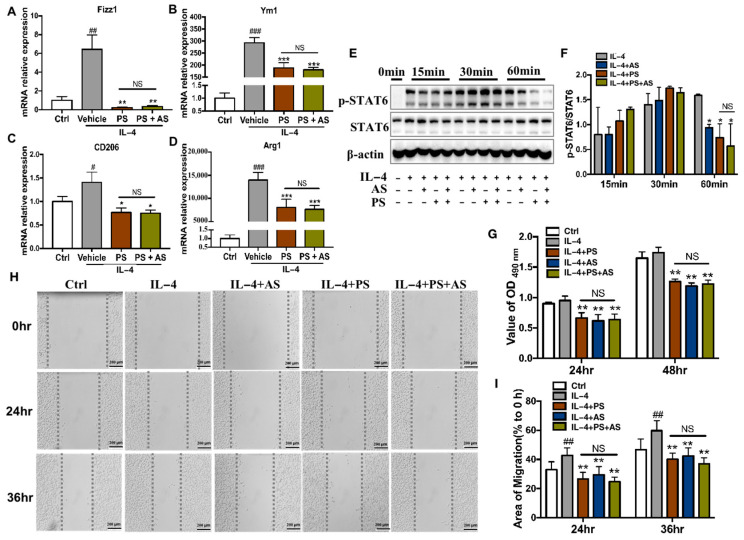
The effect of Pulsatilla saponins on M2 macrophage can be blocked by STAT6 inhibitor AS1517499. (**A**–**D**) The mRNA levels of Ym1, CD206, Fizz1, and Arg1, were detected by real-time qPCR. (**E**) Western blot analysis of STAT6 and phosphor-STAT6 in BMDM treated with vehicle or IL-4 and AS, PS, or AS+PS. (**F**) The phosphor-STAT6 protein quantified with STAT6 normalization. (**G**) B16-F0 cells were cultured in different conditioned mediums, and cell proliferation was detected by MTT assay. (**H**) Representative images for wound-healing assays and data quantification. Images were captured at 0, 24, and 36 h. (**I**) Quantification for wound-healing assay shown. Data were represented as mean ± SD, # *p* < 0.05, ## *p* < 0.01, ### *p* < 0.001, compared with the Ctrl group. * *p* < 0.05, ** *p* < 0.01, *** *p* < 0.001 compared with the vehicle (IL-4) group. “NS” represents no statistical difference. All experiments were repeated 3 times.

AS1517499 (AS), a specific inhibitor of STAT6, can inhibit STAT6 activation induced by IL-4, which is involved in the polarization of macrophages towards the M2 phenotype. This results in the suppression of M2 gene expression, including Arg1, Fizz1, Ym1, and CD206, and then alleviates the progression of tumors in vitro and in vivo [30,31,32]. To further confirm that PS inhibits macrophage M2 polarization through the IL-4/STAT6 pathway, we first compared the effect of PS on M2 gene expression with or without the STAT6 inhibitor (AS151749, AS). BMDMs were pretreated with PS (5 μg/mL) or the vehicle (control) for 12 h, then treated with 250 nM AS1517499 for 0.5 h, followed by stimulation with IL-4 (25 ng/mL) for 6 h. The results showed that PS or PS+AS significantly inhibited M2 marker genes Fizz1, Ym1, CD206, and Arg1 transcription. However, there was no significant difference between the PS and PS+AS groups (Figure 3A–D).

Further experiments using Western blot showed that PS significantly down-regulated the expression of the p-STAT6 protein in BMDM cells at 60 min of IL-4 stimulation, but not at 15 or 30 min. Pretreatment with AS or PS suppressed STAT6 phosphorylation at 60 min, and the AS+PS combination treatment did not have an additive effect. These results suggest that the inhibitory effects of PS on IL-4-induced M2 macrophage polarization depend on the STAT6 signaling pathway.

To confirm that the inhibitory effect of PS on macrophage M2 polarization is completely dependent on its inhibition of STAT6 activation, we compared the inhibitory effect of PS with that of PS+AS. CM from non-stimulated (Ctrl), IL-4-stimulated (IL-4), and IL-4 stimulation with AS (IL-4+AS), PS (IL-4+PS), or PS+AS (IL-4+AS+PS) pretreatment were collected. The proliferation and migration of B16-F0 cells were examined after treatment with different CM. The results showed that the inhibitory effect of PS on macrophage M2 polarization is comparable to that of AS, and that the combination of AS and PS does not have a superimposed inhibitory effect on macrophage M2 polarization. (Figure 3H,I). Therefore, PS achieves the inhibition of macrophage M2 polarization via a STAT6-dependent manner.

### 2.4. PS Reduces B16-F0 Melanoma Lung Metastasis by Inhibiting Macrophage M2 Polarization In Vivo

**Figure 4:**
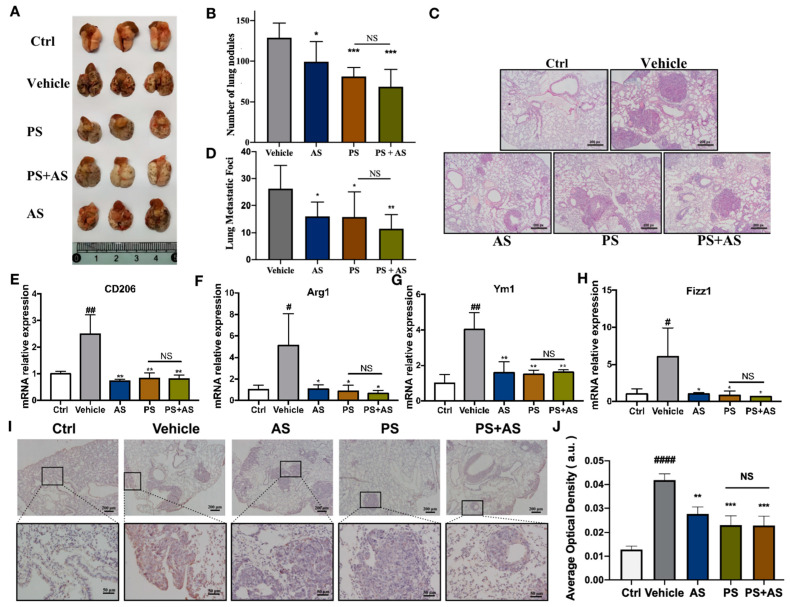
Pulsatilla saponins can inhibit B16-F0 cells lung metastasis in C57BL/6 mice, which can be blocked by STAT6 inhibitor AS1517499. (**A**) Representative macroscopic images of lung tumor metastasis points in B16-F0-bearing mice. (**B**) Quantification of metastatic numbers is shown (n = 8). (**C**) Representative H&E images of mice lungs in B16-F0-bearing mice. (**D**) Quantification of metastatic foci was shown (n = 8). (**E**–**H**) M2-related genes Arg1, Fizz1, Ym1, CD206 mRNA of lung tissue in B16-F0-bearing mice were detected by qRT-PCR (n = 3). (**I**) CD206 in lung tissue was analyzed by immunohistochemistry (n = 10). (**J**) CD206 quantification was shown. # *p* < 0.05, ## *p* < 0.01, #### *p* < 0.0001 compared with Ctrl group; * *p* < 0.05, ** *p* < 0.01, *** *p* < 0.001 compared with vehicle group. “NS” represents no statistical difference.

To examine whether PS can inhibit the development of tumors by inhibiting the polarization of M2 macrophages in vivo, we injected B16-F0 cells into C57BL/6 mice via the tail vein to establish a lung metastasis animal model. Tumor-bearing mice were treated with the vehicle, PS, AS, or PS+AS, respectively. During the entire experiment period, mice had a normal weight-increasing tendency, and there was no significant difference in the body weight of the mice in each group (Figure S2). Lung metastasis points in the PS- and AS-treated groups were significantly reduced compared with those in the vehicle-treated groups, and there was no significant difference between the PS and AS groups. There was no additive effect in the PA+AS group, compared with the AS or PS treatment (Figure 4A,B). A lung metastasis foci calculation by histopathology examination after H&E staining also confirmed these findings (Figure 4C,D). These results indicate that AS, PS, and PS+AS can effectively inhibit tumor progression in vivo, and no significant difference in tumor inhibition was observed among PS or AS alone and PS+AS in combination. This suggests that PS and AS may inhibit tumor progression through the same target, which further indicates that the tumor-inhibitory effect of PS in vivo may be due to the inhibition action of STAT6 signaling. To examine whether PS suppresses TAM polarization in vivo, the tumor-bearing lung tissue from each treatment group was freshly isolated. The RT-PCR analysis showed that PS or AS treatment significantly down-regulated M2 macrophage-specific genes Arg1, Fizz1, Ym1, and CD206 expression compared with the vehicle group (Figure 4E–H), suggesting PS inhibits alternative TAM activation (M2) in vivo. There was no difference among the AS, PS, and PS+AS treatment groups. Furthermore, the immunohistochemistry of lung tissue for CD206 showed that PS significantly suppressed CD206 expression (Figure 4I,J). Taken together, these data indicated that PS inhibited M2 polarization and attenuated B16-F0 melanoma lung metastasis in a STAT6-dependent manner.

## 3. Discussion

M2-like polarized TAMs facilitate cancer progression and metastasis by promoting tumor cell proliferation, matrix remodeling, angiogenesis, and immunosuppression [33]. Switching TAMs towards an anti-tumor phenotype has become an attractive approach to enhance the effect of chemotherapy, immunotherapy, and other treatments [34]. The current study demonstrated that PS is a critical regulator suppressing M2 macrophage polarization via the STAT6 signaling pathway in vitro and in vivo. PS inhibits tumor cell proliferation and metastasis. Our data revealed a novel mechanism for the anti-tumor activity of PS. Macrophages have a solid ability to adapt to sudden environmental changes [35]. Macrophages can be polarized into two distinct phenotypes based on their response to IFNγ/LPS (M1 macrophages) or IL-4/IL-13 (M2 macrophages) [36]. TAMs are typically M2-like and polarized by immunosuppressive cytokines such as IL-4, IL-13, or IL-10 in the tumor microenvironment. IL-4 binds to its receptor IL-4Ra, then activates the STAT6 signaling pathway, which results in the translocation of pSTAT6 to the nuclei. Once located in the nucleus, STAT6 promotes the transcription of target genes specific to M2 macrophages, such as Arg1, Fizz1, Ym1, and CD206 [37,38]. Our results showed the mRNA levels of Arg1, Fizz1, Ym1, and CD206 were increased after IL-4 stimulation, which indicated that M2 macrophage polarization was successfully induced by IL-4 stimulation. PS treatment decreased the mRNA levels of these M2 marker genes (Figure 1A–D and Figure 3A–D), suggesting PS has the activity of inhibiting M2 macrophage polarization. Since it is well-documented that M2-like TAM promotes tumor progression and metastasis through paracrine effects [27], the conditioned medium (CM) from BMDM receiving different treatments was used to culture B16-F0 cells to assess proliferation and migration activity. Consistent with previous studies [39,40,41], our data showed the CM from macrophage with IL-4 stimulation promoted the migration of B16-F0 cells, and the CM from IL-4+PS treatment inhibited B16-F0 cell proliferation (Figure 2A) and migration (Figure 2B,C). It predicated that PS could inhibit the proliferation and migration of tumor cells by affecting the polarization of M2 macrophages.

The STAT6 signaling pathway is highly activated in many tumors and has been shown to promote tumor metastasis in colorectal cancer and melanoma carcinoma [42,43,44]. The activation of STAT6 is also required for M2 macrophage polarization [45], which is reflected by the increased M2 gene expression in Stat6-overexpressing macrophages, whereas STAT6 ablation decreases M2 gene expression [46,47]. Since TAM exhibits pro-tumorigenic effects, STAT6 inhibition in TAMs might inhibit their pro-tumorigenic phenotype. Published studies have suggested that STAT6 inhibition in TAMs by AS leads to reduced TAM differentiation and thereby their pro-tumorigenic activities [48]. Genetic deletion or pharmacological inhibition of STAT6 facilitated the development of potent anti-tumor immunity based on decreasing M2 macrophage polarization, demonstrating that STAT6 in CD11b+ cells promoted lung cancer progression by increasing M2 myeloid cells in STAT6−/− mice, and the mobilization and differentiation of CD11b+ cells into M2 macrophages were decreased. Tumor growth was also inhibited [49]. In addition, there is evidence that STAT6 can be activated by IL-4 and IL-13 produced by oncogenic Kras driving cells in the tumor microenvironment, and then target cMyc to drive metabolic reprogramming. Our study showed that activated STAT6 by IL-4 promoted M2 macrophage polarization, contributing to the migration of B16-F0 cells in vitro. PS decreased STAT6 phosphorylation and nucleus translocation (Figure 1E–G). We thus hypothesized that PS might exhibit anti-tumor effects by inhibiting M2-like polarization through the STAT6 pathway.

To prove this, the target gene or protein was usually interrupted by an inhibitor/agonist, or knockdown/over-expressing [50,51]. AS, a STAT6-specific inhibitor, inhibits IL-4-induced STAT6 activation in vitro and in vivo, thereby attenuating macrophage M2 polarization [52,53] and number [54]. AS was demonstrated by a decrease in Arg1, Fizz1, Ym1, and CD206 [55], which can attenuate the progression of tumors in vitro and in vivo. It has been reported that AS can inhibit tumor growth and early liver metastasis in a 4T1 mammary carcinoma mouse model by inhibiting TAM-induced pro-tumorigenic and pro-metastatic activities via STAT6 [48]. In this study, our results also showed that AS reduced STAT6 activity (Figure 3E) and suppressed B16-F0 cell proliferation (Figure 3G) and migration (Figure 3H,I). Furthermore, PS and AS had a similar effect on inhibiting STAT6 activation, as well as M2 polarization and B16-F0 cell proliferation and migration. Importantly, we found that, compared with PS alone, the combination of PS and AS did not have an additional effect (Figure 3). Since AS is a STAT6-specific inhibitor, when STAT6 has been blocked, PS will not work if it targets STAT6. These results showed that PS+AS could not further reduce the level of phosphor-STAT6, indicating that PS acts at the same target as AS. These data indicated that the inhibiting effects of PS on M2 macrophage polarization depended on the STAT6 signaling pathway.

To further support the conclusion above, a well-established B16-F0-bearing mouse model of tumor metastasis was utilized to investigate the anti-tumor effects of PS. The results showed no significant differences in body weight among the groups with or without tumor metastasis (Figure S2). However, PS treatment significantly reduced lung metastasis (Figure 4A–D) and M2 macrophage expression (Figure 4E–J) in B16-F0 cells. Consistent with the in vitro results, combining a STAT6 inhibitor (AS) with PS had no significant difference in reducing lung metastasis and M2 macrophage expression in B16-F0 cells compared to PS treatment alone. These results indicated that PS attenuated B16-F0 tumor progression by inhibiting M2-like macrophage polarization through the STAT6 pathway.

**Figure 5:**
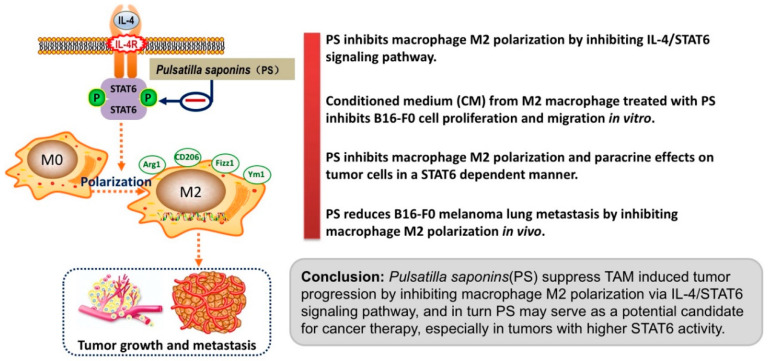
PS inhibits macrophage M2 polarization via IL-4/STAT6 signaling pathway, and in turn suppresses TAM-induced tumor progression.

In the present study, we demonstrated a novel mechanism for the anti-tumor activity of the saponin fraction of Pulsatilla chinensis (Bge.) Regel (PS), as shown in Figure 5.

In this study, PS was found to have the potential to act as a STAT6 inhibitor and effectively inhibit M2-type macrophage polarization. However, the exact mechanism of PS working against STAT6 downstream is still unknown. Since STAT6 regulates numerous genes and proteins, more research is needed to establish the direct and precise mechanism of PS against STAT6. Additionally, this study highlights that PS has a potential anti-tumor effect in cancer therapy. However, this claim needs more scientific evidence as more research is required to establish the precise effect of PS in treating cancer.

## 4. Materials and Methods

### 4.1. Antibodies

Antibody against STAT6 (Abcam, ab32520), p-STAT6 (Invitrogen, RK239130), Histone 3 antibody (CST, 4499s), β-actin (Invitrogen, MA5-15739), CD206 (Proteintech, 60143-1-Ig); horseradish-labeled goat anti-mouse IgG antibody (Zhongsu Jinqiao, ZB-2305) and horseradish-labeled goat anti-rabbit IgG antibody (Zhongsu Jinqiao, ZB-2301) were diluted at 1:1000.

### 4.2. Preparation and Identification of Pulsatilla Saponins

#### 4.2.1. Preparation of Pulsatilla Saponins

Dried Pulsatilla medicinal were materials refluxed with a 10-fold volume of 70% ethanol 3 times, 1 h for each time; the effluent was reacted with 1.0% NaOH aqueous solution for alkali hydrolysis for 6 h, then left to cool overnight, adjusted to pH 7.0 with hydrochloric acid, and applied to a D-101 microporous resin column, followed by eluting with water, 40% ethanol, 75% ethanol, and 95% ethanol in sequence. The 75% ethanol eluate was collected and concentrated under reduced pressure, then dried under vacuum conditions; *Pulsatilla Saponins* powder was prepared. This process was used both on a small scale and in a pilot-scale production, and we obtained stable and controllable *Pulsatilla Saponins* based on this process.

#### 4.2.2. Identification of Constituents of PS

To identify the constituents of Pulsatilla Saponins, the extract was screened on a Triple TOFTM 5600+ system with a Duo Spray source (AB SCIEX, Foster City, CA, USA). Briefly, the parameters’ negative ion mode was set as follows: ion spray voltage, −4500 V; ion source temperature, 550 °C; curtain gas, 30 psi; nebulizer gas (GS1), 50 psi; heater gas (GS2), 50 psi; and decluster potential (DP), −100 V. The mass ranges of TOF-MS and TOF-MS/MS experiments were all set at 50–1250 Da. In the TOF-MS/MS experiment, the most intensive eight ions from each TOF-MS scan were selected as MS/MS fragmentation. The collision energy (CE) was set at −35 eV, and the collision energy spread (CES) was (±) 15eV for the UHPLC-QTOF-MS/MS detection. The mobile phase was composed of water containing 0.1% formic acid (solvent A, *v/v*) and acetonitrile (solvent B) at a flow rate of 0.3 mL/min. A 15 min binary gradient elution as follows was performed for the separation: 0–3 min, 1% → 67% solvent B; 3–9 min, 67% solvent B; 9–12 min, 67% → 90% solvent B; 12.01–15 min, 1% solvent B; 12.01–15.0 min, followed by 3 min of column re-equilibration. In total, ten compounds were identified. The TIC profiles of those compounds are revealed in Supplementary Figure S1. Detailed information on the chemical compounds is shown in Supplementary Table S1.

### 4.3. Animals

Six- to eight-week-old male C57BL/6 mice (18–22 g) were housed in the individually ventilated cages (IVC) system under moderate temperature and humidity in 12 h light/dark conditions with free access to water. The animal study protocol was carried out in accordance with the recommendations and guidelines issued by the Animal Ethics Committee of the Jiangxi University of Traditional Chinese Medicine (Permit Number: JZLLSC2019-0353).

### 4.4. Experimental Lung Metastasis Mice Model

**Figure 6:**
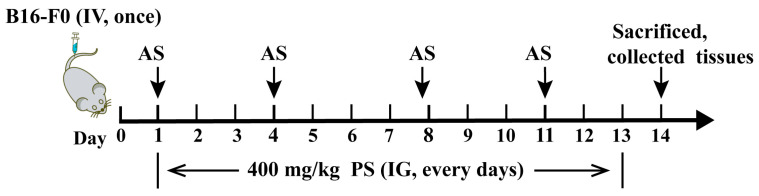
Schematic view of experimental lung metastasis mice model and treatment regimen.

The mouse lung metastasis model was established as described previously [56], and 2.5 × 10^5^ B16-F0 melanoma cells were injected into C57BL/6 mice intravenously via the tail vein. All tumor-bearing mice were divided into four groups (14 mice for each group): vehicle (treated with saline by intragastric administration), STAT6-specific inhibitor AS1517499 (MCE) (treated with AS by intraperitoneal injection twice a week at 5 mg/kg dose), PS (treated with PS by intragastric administration at 400 mg/kg once a day for 13 days) [23,25], and AS+PS (treated with AS and PS using the same dose and times with a single treatment group). All treatments were started on day 2 of the B16-F0 cell injection. The other 10 normal mice (who only received saline injections or intragastric administration) were set as the control group. Two weeks after B16-F0 cell injection, mice were sacrificed, and the lung metastasis of tumor cells was determined by quantifying the melanoma nodules in the perfused lung from 8 mice in each group. Lung tissues from the other 6 mice in each group were removed and immediately snap-frozen in liquid nitrogen for RNA or protein isolation. The experimental procedure is shown in Figure 6.

### 4.5. Cell Culture

L-929 and B16-F0 cell lines were purchased from the Chinese Academy of Sciences cell bank. Cells were maintained in RPMI1640 (Solarbio, 10491) medium supplemented with 10% fetal bovine serum (FBS, Solarbio, P1020-500), penicillin (100 U/mL), and streptomycin sulfate (100 μg/mL) in a humidified atmosphere of 5% CO_2_ at 37 °C. The L-929 supernatant was collected after 5 d 100% confluence for BMDM culture.

### 4.6. Primary Bone Marrow-Derived Macrophage (BMDM) Culture and Differentiation

BMDMs were isolated from 6-week-old male C57BL/6J mice as previously described [57]. Briefly, femurs of the mice were isolated after sacrificing by cervical dislocation, and marrow was obtained by irrigation with culture medium and dispersed by passing dispersions through a 25-gauge needle. The cells were cultured with RPMI1640 containing 10% FBS in 100 mm Petri dishes at 37 °C in a humidified 5% CO_2_ atmosphere for 3 days, then the suspension cells were collected, and these cells were maintained in RPMI1640 containing 10% FBS and 20% L-929 supernatant for 7 days to differentiate into macrophages.

### 4.7. Real-Time Quantitative PCR

Total RNAs were extracted from cells or lung tissue using the Trizol (Ambion,127703) reagent according to the manufacturer’s instructions. cDNA was synthesized using the Revert Aid First Strand cDNA Synthesis Kit (Thermo Fisher, K1622). The Real-Time PCR Kit (Applied biosystems, A25742) reactions were run in duplicate for each sample, and transcript levels of target genes were normalized to that of β-actin. The primer sequences used are shown in Table 1.

### 4.8. Preparation of Conditioned Medium

The differentiated BMDMs were treated with PS (5 μg/mL), AS (250 nM), PS (5 μg/mL) + AS (250 nM), or vehicle separately while stimulated with IL-4 (PeproTech, 96214145) (25 ng/mL) for 24 h. Then the medium was removed, and cells were washed 3 times with 1 × PBS (Solarbio, Beijing, China, P1020-500) thoroughly. The new RPMI 1640 complete medium without cytokines and drugs was added, and the conditioned medium (CM) from different groups was collected separately after 24 h incubation. The CM from BMDM cells that did not receive any IL-4, PS, or AS treatment was collected as the Ctrl group.

### 4.9. Cell Proliferation

B16-F0 cell proliferation was measured by Thiazolyl blue tetrazolium bromide (MTT) (Solarbio, M8180). Briefly, B16-F0 (3000, 7000 cells/well) was cultured in 96-well plates for 12 h, then the medium was removed, and the CM was added for 24 h or 48 h. MTT stock solutions (20 μL, 5 mg/mL in PBS) were added to the wells at the indicated time points, incubated at 37 °C for 4 h, then 150 μL DMSO (Solarbio, D8370) was added to dissolve the crystals, measured at 490 nm.

### 4.10. Wound-Healing Assay

B16-F0 cells were cultured with a starvation medium in 6-well plates for 24 h and scraped with a sterile 200 μL pipette tip. The cells were then treated with different CM for 24 or 36 h. Images were captured by using an inverted microscope at 0, 24, and 36 h. Quantification was performed by using ImageJ 1.52k software.

### 4.11. Western Blotting

A Western blot analysis was performed according to the standard protocol. Briefly, cells or lung tissue were lysed with a RIPA lysis buffer (Kaki Biology, KGP703), incubated for 30 min on ice, and centrifuged at 10,000 rpm for 5 min at 4 °C. Protein concentrations were determined using a BCA Protein Assay Kit according to the manufacturer’s instructions. Cell extracts that contained equal protein concentration were separated by 8% SDS-polyacrylamide gel electrophoresis, and then transferred to PVDF membranes. The membranes were blocked with 5% skim milk in the TBST buffer at room temperature for 1 h, then incubated with a primary antibody diluted in blocking buffer overnight at 4 °C, followed by incubation with horseradish peroxidase-conjugated secondary antibody for 1 h at room temperature. Proteins were detected by using an enhanced chemiluminescence substrate. Target protein levels were quantified by using ImageJ software.

### 4.12. Hematoxylin and Eosin Staining

Eight mice in each group were deeply anaesthetized, followed by lung perfusion via the right ventricle puncture with 4% paraformaldehyde, and then the lung tissues were removed and post-fixed in 4% paraformaldehyde for 24 h. The lung tissues were dehydrated, and paraffin embedded, then sectioned into 4–5 μm thick. Hematoxylin (Nanchang Yulu Experimental Equipment Co., Ltd., Harris A) and Eosin (Solarbio, E8090) (H&E) staining was carried out according to a standard protocol. The sections were observed and photographed using a microscope, and the lung metastases foci in the visual field were counted.

### 4.13. Immunohistochemical Staining

The lung tissue was fixed in a 4% paraformaldehyde followed by paraffin embedding. The tissue wax blocks were cut into 5 μm slices by a tissue slicer. Sections of 5 μm were deparaffinized in xylene and rehydrated in graded alcohol. Endogenous peroxidase was quenched with 0.01M sodium citrate buffer. A CD206 monoclonal antibody (Proteintech, 60143-1-Ig) was used as the primary antibody, which was detected with the 3,3′-Diaminobenzidine (DAB) Kit (Cwbio, CW2069S) according to the manufacturer’s protocol. The sections were observed and photographed using a microscope and analyzad by ImageJ software.

### 4.14. Statistical Analysis

Statistically significant differences between groups were assessed using the GraphPad Prism 8.0 software (GraphPad Software, San Diego, CA, USA). P values were calculated using the two-tailed unpaired Student’s t-test from triplicated independent experiments. Data are presented as mean ± standard deviation (SD). Two levels of significance (* *p* < 0.05, ** *p* < 0.01, *** *p* < 0.001) were used for all tests.

## 5. Conclusions

This study illustrated that PS effectively suppress tumor progression and metastasis by inhibiting the polarization of M2 macrophages via the STAT6 signaling pathway, a novel mechanism of PS’s anti-tumor activity.

## Figures and Tables

**Table 1:** The primer sequences for RT-PCR.

Gene Name	Sense 5′-3′	Antisense 5′-3′
β-actin	ATGACCCAGATCATGTTTGA	TACGACCAGAGGCATACAG
Ym1	TCTCTACTCCTCAGAACCGTCAGA	GATGTTTGTCCTTAGGAGGGCTTC
CD206	ACGAGCAGGTGCAGTTTACA	GCTGCATTGGAGAGGTGTCT
Fizz1	TACTTGCAACTGCCTGTGCTTACT	TATCAAAGCTGGGTTCTCCACCTC
Arg1	CAAGGTGATGGAAGAGACCTT	TAAGGTAGTCAGTCCCTGGCTT

## Data Availability

Not applicable.

## Supplementary Materials

The following supporting information can be downloaded at: https://www.mdpi.com/article/10.3390/molecules28093682/s1, Figure S1: PS extraction ion chromatogram obtained by UPLC-Q-TOF-MS/MS; Table S1: Constituents Identification of Pulsatilla Saponins obtained by UPLC-Q-TOF-MS/MS; Figure S2: Pulsatilla saponins (PS) had no effect on body weight in mice with lung metastases.
