# Supplementary material for: Pulsatilla Saponins Inhibit Experimental Lung Metastasis of Melanoma via Targeting STAT6-Mediated M2 Macrophages Polarization
Source: Molecules. 2023 Apr 24;28(9):3682. doi: 10.3390/molecules28093682 (PMC10179893; doi:10.3390/molecules28093682)
Supplement: Supplementary file 1 [file molecules-28-03682-s001.zip › molecules-2305991-supplementary.pdf]

molecules

MDPI

Articl

Pulsatilla saponins inhibit experimental lung metastasis of melanoma via targeting STAT6-mediated M2 macrophages polarization

Xin Yang[1,†], Miaolin Wu[1,†], Xin Yan[2], Cheng Zhang[3], Yingying Luo[1,3,4,*], Jun Yu[3,*]

1 Center for Translational Medicine, Jiangxi Key Laboratory of Traditional Chinese Medicine in Prevention and Treatment of Vascular Remodeling Associated Disease, Jiangxi University of Chinese Medicine, Nanchang 330006, China
2 The Second Affiliated Hospital, Jiangxi University of Chinese Medicine, Nanchang 330006, China
3 Department of Cardiovascular Sciences and Center for Metabolic Disease Research, Lewis Katz School of Medicine, Temple University, Philadelphia, PA 19140, USA
4 State Key Laboratory of Innovative Drug and Efficient Energy-Saving Pharmaceutical Equipment, No. 56 Yangming Road, Nanchang 330006, PR China
* Correspondence: luoying0302@163.com (Y.L.); jun.yu@temple.edu (J.Y.); Tel.: +86-791-87119615 (Y.L.)
† These authors contributed equally to this work.

Citation: To be added by editorial staff during production.

Academic Editor: Firstname Lastname

Received: date
Revised: date
Accepted: date
Published: date

Copyright: © 2023 by the authors. Submitted for possible open access publication under the terms and conditions of the Creative Commons Attribution (CC BY) license (https://creativecommons.org/licenses/by/4.0/).

Supplementary Figure S1

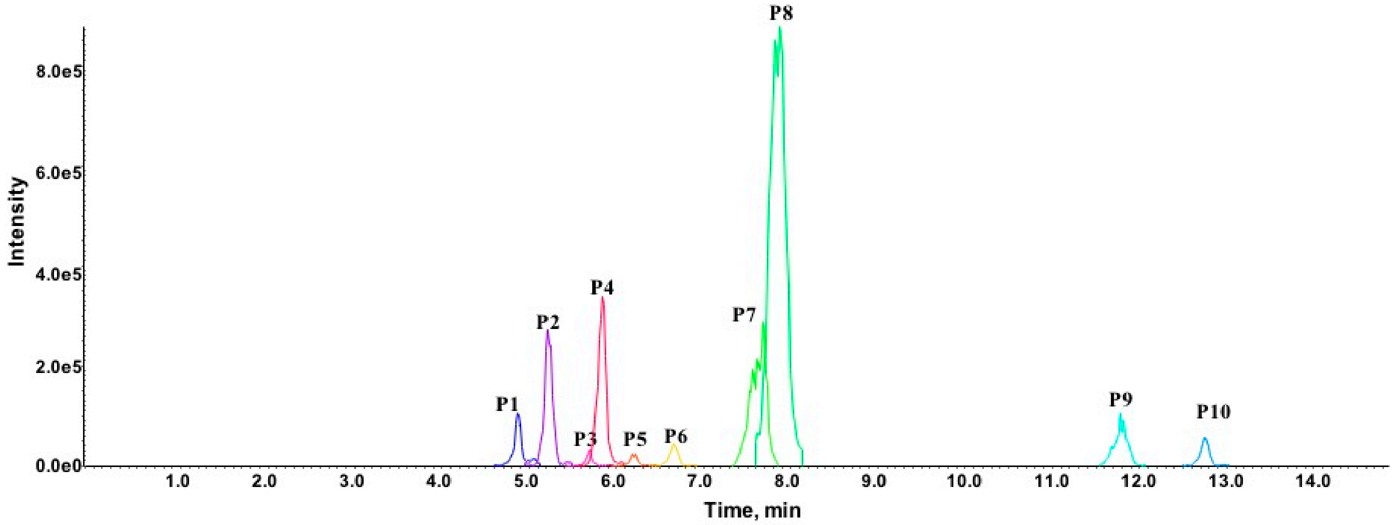

Figure S1. PS extraction ion chromatogram obtained by UPLC-Q-TOF-MS/MS.

Supplementary Table S1.

Table S1. Constituents Identification of *Pulsatilla Saponins* obtained by UPLC-Q-TOF-MS/MS

NO.	tR（min）	Formula	Calculated [M-H]-m/z	Experimental [M-H]-m/z	Error (ppm)	（MS2）m/z	Relative areas ×10^5	Identification
P1	4.97	C53H86O22	1073.5338	1073.56203	0.7	911.5133,603.3945	1.0	Hederacoside D
P2	5.32	C47H76O17	911.50098	911.50761	0.3	749.4578,603.3956,471.3509	2.7	Pulsatilla saponin D
P3	5.79	C41H66O13	765.44307	765.44894	1.2	603.3945,471.3503	3.2	Hederagenin sapogenin 3-O-β-D-glucopyranosyl-(1→4)-α-L-arabinose
P4	5.93	C47H76O17	911.50098	911.50812	0.9	749.4578,603.3956,471.3509	3.4	Hederagenin sapogenin 3-O-β-D-glucopyranosyl-(1→3)-α-L-rhamnopyranosyl-(1→2)-α-L-arabinose
P5	6.30	C41H66O12	749.44815	749.45188	5	603.3887,471.3510	2.3	Hederagenin sapogenin 3-O-α-L-rhamnopyranosyl-(1→2)-α-L-arabinose
P6	6.76	C48H78O18	941.51154	941.5183	2.2	895.5181,733.4625,455.3553	4.4	Pulsatilloside C
P7	7.69	C47H76O16	895.50606	895.51398	0.8	733.4619,587.4006,455.3553	2.9	Oleanolic acid 3-O-β-D-glucopyranosyl-(1→4)-[α-L-rhamnanopyranosyl-(1→2)]-α-L-arabinose
P8	7.96	C53H86O21	1057.55888	1057.56851	3.8	733.4614,587.4001,455.3549	8.8	Oleanolic acid 3-O-β-D-glucopyranosyl-(1→4)-β-D-glucopyranosyl-(1→3)-α-L-rhamnanopyranosyl-(1→2)-α-L-arabinopyranoside
P9	11.9	C41H66O11	733.45324	733.45954	1.2	569.3900，455.3558	10.0	Oleanolic acid 3-O-α-L-rhamnopyranosyl-(1->2)-α-L-arabinopyranoside
P10	12.85	C30H48O4	471.34798	471.35029	1.3	393.3183，333.2231	5.6	Hederagenin

Supplementary Figure S2

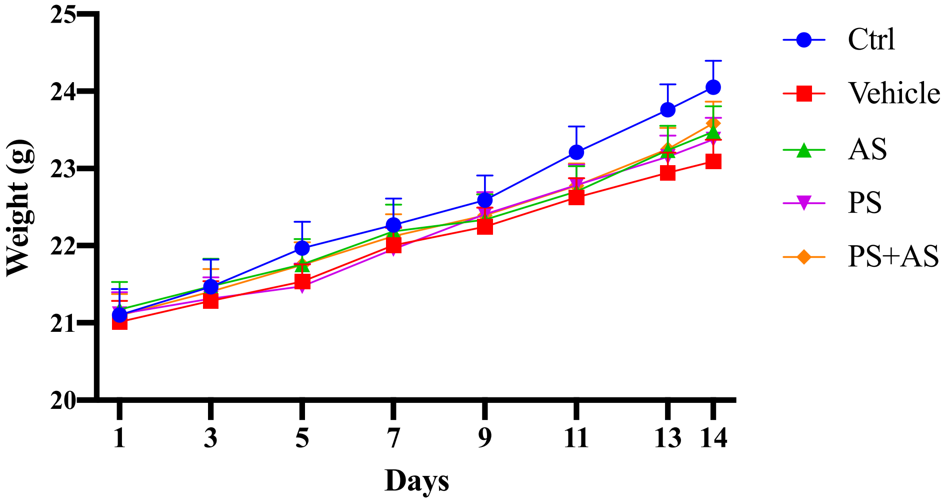

Supplementary Figure S2. Pulsatilla saponins (PS) had no effect on body weight in mice with lung metastases. Weight change map of mice.
